# Mismatched single stranded antisense oligonucleotides can induce efficient dystrophin splice switching

**DOI:** 10.1186/1471-2350-12-141

**Published:** 2011-10-20

**Authors:** Clayton T Fragall, Abbie M Adams, Russell D Johnsen, Ryszard Kole, Sue Fletcher, Steve D Wilton

**Affiliations:** 1 Centre for Neuromuscular and Neurological Disorders, University of Western Australia, 35 Stirling Highway, Crawley, Western Australia 6009, Australia; 2 AVI Biopharma, 3450 Monte Villa Parkway, Bothell, WA 98021, USA

## Abstract

**Background:**

Antisense oligomer induced exon skipping aims to reduce the severity of Duchenne muscular dystrophy by redirecting splicing during pre-RNA processing such that the causative mutation is by-passed and a shorter but partially functional Becker muscular dystrophy-like dystrophin isoform is produced. Normal exons are generally targeted to restore the dystrophin reading frame however, an appreciable subset of dystrophin mutations are intra-exonic and therefore have the potential to compromise oligomer efficiency, necessitating personalised oligomer design for some patients. Although antisense oligomers are easily personalised, it remains unclear whether all patient polymorphisms within antisense oligomer target sequences will require the costly process of producing and validating patient specific compounds.

**Methods:**

Here we report preclinical testing of a panel of splice switching antisense oligomers, designed to excise exon 25 from the dystrophin transcript, in normal and dystrophic patient cells. These patient cells harbour a single base insertion in exon 25 that lies within the target sequence of an oligomer shown to be effective at removing exon 25.

**Results:**

It was anticipated that such a mutation would compromise oligomer binding and efficiency. However, we show that, despite the mismatch an oligomer, designed and optimised to excise exon 25 from the normal dystrophin mRNA, removes the mutated exon 25 more efficiently than the mutation-specific oligomer.

**Conclusion:**

This raises the possibility that mismatched AOs could still be therapeutically applicable in some cases, negating the necessity to produce patient-specific compounds.

## Background

Antisense oligomer (AO) induced exon skipping has emerged as a promising approach to reduce the severity of Duchenne Muscular Dystrophy (DMD), progressing rapidly from concept to the completion of several clinical trials [1-4]. This therapy uses AOs to modify splicing during pre-RNA processing, such that a DMD-associated exon is removed and a shorter but partially functional Becker muscular dystrophy (BMD)-like dystrophin isoform is produced. Most commonly, splice switching AOs are designed to target motifs in the normal dystrophin gene transcript, which is appropriate, since the most common type of DMD mutation is a deletion of one or more exons and it is the normal exon, flanking the deletion that must be removed to restore the reading frame. In addition, it may be assumed that normal exons will generally be more difficult to dislodge than a mutated counter-part, since the full complement of splicing motifs will be present in the former.

Disease-causing gene lesions, silent polymorphisms or small intra-exonic deletions, insertions or substitutions may occur within the oligomer annealing region, or impact upon splice control motifs. These could potentially alter the efficacy of an AO designed and optimised for the normal dystrophin gene transcripts. Data from the Human Genome Project suggests that single nucleotide polymorphisms (SNPs) occur every 100-300 bases http://www.ornl.gov/sci/techresources/Human_Genome/faq/snps.shtml. Although the distribution of these SNPs is non-random, their incidence means that patients may harbour a SNP in the target exon. Given that dystrophin exons are, on average, only 150 bp long and splice switching AOs are designed as 25-32mers, patients carrying intra-exonic disease-causing mutations will have an approximately 1 in 6 chance that the gene lesion will occur within the AO annealing sequence. Consequently, even DMD boys with the "same" mutation at the mRNA level (e.g. a genomic deletion of exon 50) will almost certainly have unique dystrophin genes because of differences in disease-associated deletion break-points, as well as non-disease associated DNA variations in both protein-coding and non-coding regions. It should not be unexpected that each DMD boy could respond to induced exon skipping in a unique manner. For this reason, the development of optimal exon skipping strategies for most DMD mutations are best evaluated in cells from the patient.

Subtle dystrophin gene changes, such as intra-exonic insertion/deletions/substitutions collectively represent less than 30% of cases but are spread across the dystrophin gene, making them a lower priority for immediate clinical development than amenable mutation sub-types in the deletion hotspots. In anticipation of accelerated development of oligomer induced exon skipping as a therapy for DMD, we wish to develop effective exon skipping strategies for as many different DMD mutations as possible. As described by Wilton *et al *[5], we have optimised AOs to exclude each of the exons in the dystrophin transcript, excluding the first and last exons, and are now focused upon further refining AO design during pre-clinical testing in DMD patient cell lines.

Here we present the optimization of oligomers to skip exon 25, and report the unexpected finding that a mismatched oligomer induced efficient exon exclusion in cells from a young patient, with a single base insertion in exon 25 (c.3385 Insertion A) that lies within the optimized AO target sequence.

## Methods

### AO design and synthesis

Oligomers consisting of 2'-O-methyl (2OMe) modified bases on a phosphorothioate backbone were synthesized on an Expedite 8909 synthesizer (Applied Biosystems, Melbourne, Australia), as described by Adams *et al*. [6]. AO sequences are shown in Table 1 (AO nomenclature according to Mann *et al *[7]). The optimal sequence to excise exon 25 was then prepared and supplied by AVI Biopharma Inc (Bothell, WA) as a phosphorodiamidate morpholino oligomer (PMO) conjugated to a cell-penetrating peptide (PPMO-*k*) [8,9].

**Table 1:** Oligomer Sequences

Oligomer	Sequence (5' to 3')
H25A(+10+33)	UGG GCU GAA UUG UCU GAA UAU CAC
H25A(+95+119)	UUG AGU UCU GUC UCA AGU CUC GAA G
H25A(+95+A+119)	UUG AGU UCU GU**U **CUC AAG UCU CGA AG
H25D(+16-08)	GUC UAU ACC UGU UGG CAC AUG UGA
H25D(+06-14)	GAG AUU GUC UAU ACC UGU UG

### Cell Culture and Transfection

Normal human myoblasts were prepared as described by Rando *et al*. [10] from de-identified muscle biopsies, obtained with informed consent during elective surgery in the Department of Neuropathology at Royal Perth Hospital. Similarly donated after informed consent, de-identified fibroblasts were obtained from a DMD patient harbouring a frame shifting mutation in exon 25 (c.3385 Insertion A). Fibroblasts were cultured in DMEM (Invitrogen, Melbourne, Australia) supplemented with 20% foetal calf serum (FCS) (Serana, Bunbury, Australia), 1% GlutaMax™-I (Gibco, Melbourne, Australia), 10 U/ml penicillin (Invitrogen), 10 mg/ml streptomycin (Invitrogen), and 250 ng/ml amphotericin B (Sigma, Sydney, Australia). Fibroblasts were converted to myoblasts through forced myogenesis by transfection with a MyoD expressing adenovirus [11] and then differentiated in low serum media. Briefly, patient fibroblasts were cultured to 80% confluency, washed with PBS, detached with 0.25% Trypsin (w/v) (Gibco), inactivated with media containing 10% FCS, pelleted by centrifugation at 600 × g and then resuspended in DMEM supplemented with 5% horse serum and the MyoD adenoviral vector at a multiplicity of infection of 250. Ninety six hours after treatment with the MyoD adenoviral vector the fibroblasts were transfected with AO. The use of human tissue was approved by the University of Western Australia Human Ethics Committee (approval number RA/4/1/2295).

Normal myoblasts were proliferated and differentiated as described previously by Harding *et al*. [12]. All cells were plated at 2 × 10^4 ^cells/well in 24 well plates that had been sequentially pre-treated for 1 hour with 50 μg/ml poly D-lysine (Sigma) and 100 μg/ml Matrigel (BD Biosciences, Sydney, Australia).

2OMeAOs were transfected as lipoplexes with Lipofectamine 2000^® ^(1:1 w/w) (Invitrogen) in Opti-MEM media (Gibco) as per the manufacturer's instructions. PPMO-*k *solutions were warmed for 5 minutes at 37°C before being diluted in Opti-MEM media as indicated and applied directly to the adherent cells.

### RNA extraction and nested RT-PCR

Total RNA was harvested 96 h after transfection from duplicate wells using TRIzol^® ^(Invitrogen) according to the manufacturer's instructions, and resuspended in 30 μl of sterile water (Baxter Healthcare, Sydney, Australia). Approximately 100 ng of total RNA was used as template for primary amplification using Superscript^® ^III One-step RT-PCR system with Platinum Taq (Invitrogen) to amplify exons 13 to 27. After 35 cycles (myoblasts) and 40 cycles (MyoD converted fibroblasts) a 1 μL aliquot was removed and subjected to nested PCR to amplify exons 18 to 26 for 30 cycles (myoblasts) or 35 cycles (MyoD converted fibroblasts), using AmpliTaq Gold (Applied Biosystems). Details of PCR primers (Geneworks, Adelaide, Australia) are shown in Table 2.

**Table 2:** Dystrophin primers used for nested RT-PCR

PCR	Exon	Direction	Sequence (5' to 3')
Primary	13	Forward	AGC TTC AAG AAG ATC TAG AAC AAG AAC A
Primary	27	Reverse	GCT ATG ACA CTA TTT ACA GAC TC
Secondary	18	Forward	GAT ATA ACT GAA GTT CAC AG
Secondary	26	Reverse	ATT CGT GCA TCT CTG ATA GAT C

### Gel Analysis, Imaging and Sequencing

PCR products were resolved on 2% agarose gels in TAE buffer and relative exon skipping efficiency estimated by densitometry of the full length and AO induced PCR products on images captured by the Chemi-Smart 3000 system (Vilber Lourmat, Marne-la-Vallée, France) as described previously [6]. Where necessary, the identity of induced transcripts was confirmed by band stab isolation [13], purification of templates using UltraClean spin columns (MoBio, Carlsbad, CA) and DNA sequencing using BigDye V3.1 terminator chemistry (Applied Biosystems) as per manufacturer's instructions. Sequencing was conducted at the Lotterywest State Biomedical Facility Genomics (Perth, Australia).

### Western analysis of dystrophin expression

Western blotting was performed using a protocol derived from Cooper *et al *[14] and Nicholson *et al *[15]. Cells were harvested by trypsinisation, 7 days after transfection and placed in treatment buffer (100 μl/4.5 mg wet pellet weight) consisting of 125 mM Tris-HCl pH 6.8, 15% SDS, 10% glycerol, 0.5 mM PMSF, 50 mM dithiothreitol, bromophenol blue (0.004% w/v) and a protease inhibitor cocktail (15 μl/500 μl of treatment buffer) (Sigma). Samples were vortexed briefly, sonicated for 1 second, 4-8 times at a setting of 30/100 on an ultrasonic processor (Sonics, Newtown, CT) and heated at 95°C for 5 minutes. Samples were then electrophoresed at 16°C on a 3-10% Tris- Bis/Glycine SDS gradient gel at pH 8.8 with a 3% stacking gel pH 6.8. Gel contents were electrophoretically transferred to a FluorotransW PVDF membrane (Pall, Melbourne, Australia) overnight at 18°C at 290 mA in a transfer buffer without methanol. Dystrophin was detected with NCL-DYS2 monoclonal anti-dystrophin (Novocastra, Newcastle-upon-Tyne, UK) applied at a dilution of 1:100 for 2 hours at room temperature. Detection was performed using a Western Breeze kit as per the manufacturer's instructions (Invitrogen). Enhanced Chemiluminescence reactions were detected directly by the Chemi-Smart 3000 gel documentation system (Vilber Lourmat), using Chemi-Capt software for image acquisition and Bio-1D software for image analysis.

## Results

**Figure 1:**
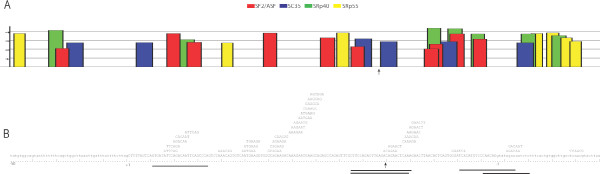
**Designing AOs to target ESEs in dystrophin exon 25**. ESE finder analysis indicates the location of potential binding sites for the splicing factors SF2/ASF, SC35, SRp40 and SRp55 in exon 25 (A). Rescue ESE analysis was employed to gauge the activity of predicted exonic splice enhancers (ESE) (B). The arrow indicates the location of the mutation (c.3385 Insertion A), although this single base insertion made no difference to the splice motifs predicted by ESE Finder (A) or Rescue ESE (B). Antisense oligonucleotide annealing positions are indicated by the solid bars below the sequence (B). The specific sequences and exonic annealing co-ordinates of these AOs can be found in Table 1.

The splice motif predictor programs, ESE finder [16], and Rescue ESE [17] were used to predict putative exonic splicing enhancers (ESEs) in exon 25 (Figure 1). Annealing co-ordinates of AOs relative to the predictive ESE positions are indicated.

**Figure 2:**
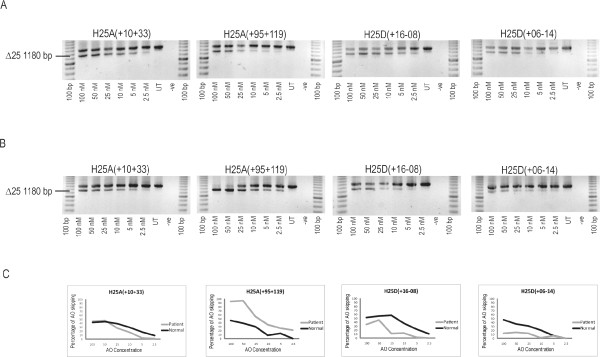
**2OMeAO mediated excision of dystrophin exon 25 in normal and patient cells**. 2OMeAOs designed to excise exon 25 from the dystrophin transcript were transfected into normal human myoblasts (A) and MyoD adenovirus converted (c.3385 Insertion A) patient myogenic cells (B). Nested RT-PCR was undertaken across exons 18-26 and densitometry used to quantify the relative levels of full length and exon 25 deleted (Δ25) amplicon in each lane as a measure of exon skipping efficacy. The percentage of exon 25 skipping induced by AOs in both normal (black line) and patient cells (grey line) is presented (C).

2OMeAOs designed to target the predicted splicing motifs in dystrophin exon 25 (Table 1 and Figure 1) were transfected into normal human myoblasts (Figure 2A). In normal cells, all AOs tested demonstrated robust skipping of exon 25 at 100 nM and were effective at the lower concentration of 2.5 nM.

Figure 2B shows the levels of exon 25 skipping in MyoD converted patient fibroblasts after treatment with the same panel of AOs. H25D(+16-08) and H25D(+06-14), targeting the exon 25 donor splice site, did not induce the same degree of exon skipping efficiency in the patient cells (Figure 2B). H25A(+10+33) induced similar levels of exon 25 excision in patient and normal cells, whereas H25A(+95+119) performed better in patient cells than in normal cells at all concentrations tested (Figure 2B and 2C).

**Figure 3:**
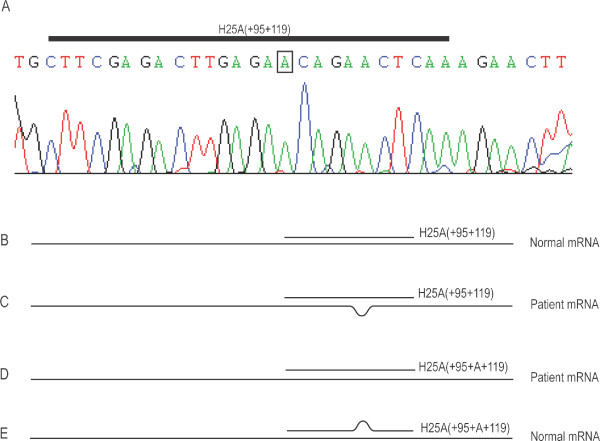
**Confirmation of the patient dystrophin mutation and the potential effect on AO Annealing**. The dystrophin (c.3385 Insertion A) mutation was confirmed by direct sequencing (A). This mutation occurs within the annealing site of H25A(+95+119) suggesting a number of potential scenarios for the binding of H25A(+95+119) or H25A(+95+A+119) including; perfect complementarity between H25A(+95+119) and normal dystrophin mRNA (B), a single base "bulge" in the mRNA when H25A(+95+119) binds to patient mRNA (C), perfect complementarity between H25A(+95+A+119) and patient mRNA (D) and a single base "bulge" in H25A(+95+A+119) when this oligomer binds to normal mRNA (E).

H25A(+95+119) targets the region of dystrophin exon 25 that encompasses the causative mutation. Since this mismatched oligomer generated such effective exon skipping, we subsequently examined the patient's mutation by direct DNA sequencing and confirmed the original diagnosis of a single base insertion of an A, 110 bases from the beginning of exon 25 (Figure 3A). This mutation occurs 11 bases downstream from the 5' end of H25A(+95+119), raising questions about how it might be influencing AO efficacy (Figure 3B and 3C). To investigate this we then designed a mutation specific AO to target the same coordinates.

**Figure 4:**
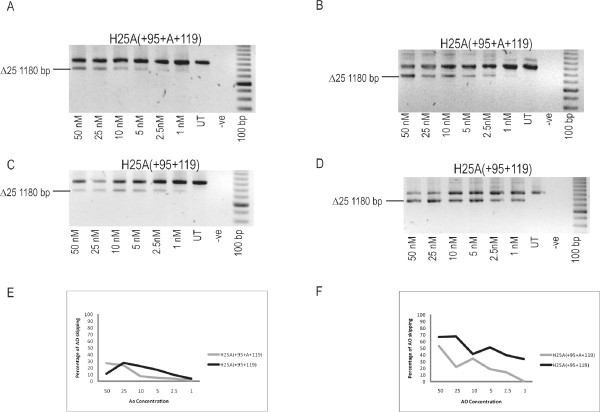
**Comparison of optimised and personalised 2OMeAOs for the excision of Exon 25 in normal and patient cells**. The 2OMeAOs H25A(+95+119) and patient-specific H25A(+95+A+119) were transfected into normal myoblasts (A, C and E) and MyoD adenovirus converted (c.3385 Insertion A) patient myoblasts (B, D and F) at the concentrations indicated. Nested RT-PCR was undertaken across exons 18-26 and densitometry used to quantify the levels of full length and exon 25-deleted (Δ25) amplicon in each lane as a relative measure of exon skipping efficiency. The percentage of exon 25 skipping induced by H25A(+95+119) (black line) and H25A(+95+A+119) (grey line) is presented graphically for normal (E) and patient cells (F).

AO H25A(+95+A+119) was produced as a patient-specific AO with perfect complementarity when annealing to the patient dystrophin transcript (Figure 3D and Table 1). Surprisingly, transfection of patient cells with H25A(+95+A+119) shows that perfect complementarity is not essential for efficient exon skipping in this case (Figure 4). Although the additional base makes little difference when compared to H25A(+95+119) in normal cells, despite the mismatch (Figure 4A), in the patient cells H25A(+95+A+119) reduces the efficiency of exon skipping, particularly evident at lower transfection concentrations (Figure 4B).

**Figure 5:**
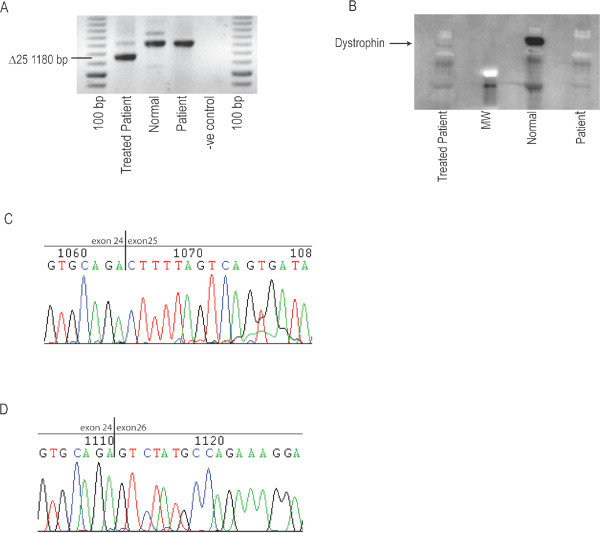
**PPMO-*k *H25A(+95+A+119) mediated excision of exon 25 from patient cells**. H25A(+95+A+119) synthesized as the PPMO-*k *chemistry was transfected into MyoD adenovirus converted patient cells (c.3385 Insertion A). The cells were collected 7 days after transfection for analysis of dystrophin mRNA (A) and protein (B) levels. Samples from normal myoblasts (Normal) and MyoD converted patient myogenic cells (Patient) are included for comparison. Sequencing of the predominant PCR products generated from the PPMO-*k *treated patient cells and control myoblasts (A) confirmed the normal exon 24/25 boundary in control myoblasts (C) and the exclusion of exon 25 in the PPMO-*k *treated patient transcripts (D).

DMD patient fibroblasts were converted to myoblasts by forced myogenesis and transfected with H25A(+95+119)-*k *(i.e. PPMO-*k *chemistry). Untreated Myo-D converted DMD fibroblasts, and normal myoblasts were used as negative and positive controls, respectively. Figure 5 shows that transfection with H25A(+95+119)-*k *at 800 nM led to significant skipping of exon 25 from the dystrophin transcript (Figure 5A), and production of dystrophin protein (Figure 5B) whereas neither could be detected in patient forced myogenic cells. Sequencing of the predominant PCR products generated from the H25A(+95+119)-*k *treated patient and control myoblasts (Figure 5A) confirmed the exclusion of exon 25 from the amplicon (Figure 5C and 5D).

## Discussion

DMD patients with whole exon deletions or duplications can potentially be treated by excising normal exons that should occur in a near-normal context. For this reason, unaffected normal human myoblasts are routinely used for the development and optimisation of splice-switching AO sequences, and we have previously shown that the AOs that are most effective in normal cells are generally also the most effective in patient cells [18]. However, we recently reported that some intra-exonic changes can influence the efficiency of AO-mediated splice manipulation [19].

A panel of AOs were designed to target human dystrophin exon 25 and when applied to patient cells carrying a single base insertion, all induced exon 25 excision. This DMD-causing gene lesion did not alter any exonic splicing motifs, as determined by *in silico *analysis, but most unexpectedly H25A(+95+119), which annealed across the insertion and was therefore mismatched, induced the most robust exon skipping. When compared to the profile of exon skipping induced in normal cells, H25A(+95+119) appeared to be about twice as effective in the patient cells at all concentrations tested. Some enhancement of exon skipping in the patient cells would be due to the now in-frame, AO induced transcripts no longer being subjected to nonsense mediated decay (NMD). Evasion of NMD would increase the mRNA half life of the transcript missing exon 25, thereby increasing its abundance in relation to the out-of-frame DMD transcript. Conversely, removal of exon 25 from the normal dystrophin gene transcript does not disrupt the reading frame, hence intact and exon 25-deleted transcripts should show similar turnover.

We can only speculate as to why the mismatched H25A(+95+119) oligomer performed so effectively in the patient cells in comparison to the mutation specific H25A(+95+A+119). Previous mismatched oligomer studies by us [20] and others [21,22], utilized sequences with mismatched bases but no insertions or deletions within the oligomer:mRNA duplex. Here, we have an oligomer annealing across a single base insertion that would presumably loop-out and/or alter the secondary structure of the pre-mRNA to further compromise exon recognition and selection. The exon 25 skipping induced by the patient-specific oligomer was comparable to that generated after transfection with the originally optimized H25A(+95+119) in normal cells. We are now re-evaluating oligomer design in our laboratory and including selected mismatches in well-studied splice switching oligomers to determine if this effect is unique to this mutation or reflects a more general mechanism (e.g. miRNA binding of seed sequences in their target gene transcripts [23]).

Testing of oligomers in patient cells will be the most appropriate way to determine if patient-specific mutations or polymorphisms compromise AO efficacy, and in the event of diminished oligomer efficacy, an alternative sequence should be evaluated. Data reported by us [20] and others [21,22] have reported that mismatched AOs compromise exon skipping efficiency and we predicted that personalizing an oligomer optimized in normal cells to match the patient sequence would further enhance efficacy. However, this proved otherwise in the case presented here. The personalized H25A(+95+A+119) induced exon skipping in both normal and patient cells but at markedly lower levels than the mismatched oligomer designed to the normal dystrophin coordinates.

Detecting dystrophin protein following induced exon skipping in DMD fibroblasts that had undergone forced myogenesis with the MyoD adenovirus can be technically challenging, and we were unable to demonstrate dystrophin by Western blotting after treatment with the 2OMe H25A(+95+119). In order to confirm that the efficacy of the mismatched oligomer was not chemistry specific, forced myogenic DMD cells were transfected with the oligomer sequence prepared as PPMO-*k*. Efficient exon skipping and detectable dystrophin protein was induced after H25A(+95+119)-*k *treatment, indicating that this effect could also be achieved with the morpholino oligomer chemistry. We did not contemplate using a customized PPMO-*k *for this mutation because of the cost of the compound and its restricted applicability.

## Conclusions

Initially, exon skipping to treat DMD will only be applied to deletions within mutation hotspots. This is despite the fact that nearly half of the dystrophin exons are in-frame and therefore many intra-exonic mutations could be addressed by single exon skipping. Although many such exons will be highly amenable targets for oligomer intervention, the incentive to pursue these targets is limited by the fact that in-frame exons do not restore commonly encountered exon deletion frame-shifts and intra-exonic mutations are spread across the gene, with no prominent hotspots. Furthermore, the presence of intra-exonic mutations or SNPs may increase or decrease exon skipping efficacy by altering splice motifs or directly compromising oligomer annealing, implying that the patients may require mutation-specific oligomer design. Toxicology and safety validation adds significant costs to bringing each AO to the clinic, and even single base changes (e.g. personalisation) to approved AO designs will require such validation. Here we show that this level of personalised medicine may not be necessary in all cases.

## Competing interests

The authors declare that they have no competing interests.

## Authors' contributions

CTF participated in data analysis and interpretation, and drafted the manuscript. AMA and RDJ were responsible for data acquisition and analysis. SF, RK and SDW conceived of the study and participated in its design, coordination and interpretation. All authors reviewed the draft documents and approved the final manuscript.

## Pre-publication history

The pre-publication history for this paper can be accessed here:

http://www.biomedcentral.com/1471-2350/12/141/prepub
